# A cost-effective data assimilation system for improving ROMS simulations in the Southwest Atlantic

**DOI:** 10.1371/journal.pone.0358351

**Published:** 2026-10-20

**Authors:** Geoff S. Dorfschäfer, Clemente A. S. Tanajura, Thiago P. De Paula, Filipe B. Costa, Janini Pereira

**Affiliations:** 1 Oceanographic Modeling and Observation Network (REMO), Salvador, Bahia, Brazil; 2 Graduate Program in Geophysics, Federal University of Bahia (UFBA), Salvador, Bahia, Brazil; 3 Department of Earth and Environmental Physics, Physics Institute, Federal University of Bahia (UFBA), Salvador, Bahia, Brazil; 4 Department of Oceanic Engineering Technology, Petrobras Research and Development Center (CENPES), Rio de Janeiro, Rio de Janeiro, Brazil; 5 Federal University of Rio de Janeiro (UFRJ), Alberto Luiz Coimbra Institute for Graduate Studies and Research in Engineering (COPPE), Rio de Janeiro, Rio de Janeiro, Brazil; 6 CMCC Foundation - Euro-Mediterranean Center on Climate Change, Lecce, Italy; Universidade de Aveiro, PORTUGAL

## Abstract

This study presents for the first time the integration of the low-cost REMO Ocean Data Assimilation System (RODAS) into the Regional Ocean Modeling System (ROMS) for assimilating Sea Surface Temperature (SST) observations. RODAS is an Ensemble Optimal Interpolation (EnOI)-based data assimilation system originally developed for use with the HYCOM model. Here, it is adapted to ROMS and applied to the Southwest Atlantic, with a focus on evaluating its impact on short-term ocean hindcasts. We conducted 4-years simulations with and without SST assimilation, referred to as RODAS_SST and Free, respectively. The RODAS_SST experiment showed a 70% reduction in SST root mean square deviation (RMSD) relative to the Free run, and a 55% reduction compared to 72-hour hindcasts generated from previous analysis cycles. When compared with the GLORYS reanalysis, SST RMSD was reduced by 64%. Ocean heat content estimates also improved, with reductions in mean absolute error of 15% during summer and 25% in winter. While assimilation improved surface and subsurface thermal structure in general, comparisons with Argo profiles revealed occasional degradations near the thermocline, highlighting the need for better multivariate covariance representation in the EnOI scheme. The successful implementation of RODAS in ROMS provides a cost-effective tool for enhancing model initial conditions and regional ocean simulations. Future developments will include the assimilation of Argo temperature and salinity profiles, sea level anomaly (SLA) data, and observations from the SWOT satellite. The code will be openly released, aiming to broaden access to ocean data assimilation by enabling robust performance even on limited computational resources.

## 1. Introduction

Sea surface temperature (SST) is one of the key variables capable of impacting oceanic and atmospheric systems on both regional and global scales, directly influencing cyclone formation [1], ocean currents [2], climate phenomena such as El Niño and La Niña [3] and the intensity of extreme events like marine heatwaves. SST data analysis contributes to the optimization of maritime routes, fishery resource management, and variations in its anomalies are particularly important for marine ecology [4]. This crucial variable helps us understand atmosphere-ocean interactions and is essential for improving the accuracy of predictions derived from numerical models [5].

Numerical models are tools that enable us to predict atmospheric, oceanic, and climatic phenomena. These models use nonlinear equations to describe state variables at specific grid points. Over the years, various models have provided different solutions for simulating the oceanic variables, such as the Hybrid Coordinate Ocean Model, HYCOM [6], the Modular Ocean Model, MOM [7], and the Nucleus for European Modelling of the Ocean, NEMO [8]. One such model is the Regional Ocean Modeling System (ROMS) [9], which is commonly used for studying and forecasting oceanic variables on a regional scale [10] or larger scales [11]. Although the model’s predictabilities show relative confidence, limitations in the initial conditions or in the approximations employed by the governing equations may result in significant errors, compromising the reliability of forecasts and oceanic simulations [12].

The accuracy of simulations in numerical models directly depends on the quality of initial conditions [13,14]. To enhance these conditions, data assimilation (DA) emerges as a method that combines observations with simulations to generate the objective analysis, the state immediately after corrections are applied. Several studies have demonstrated that DA has the potential to overcome numerical limitations and improve short-term ROMS forecasts [15–18]. Historically, a wide range of DA methods has been developed to improve model outputs. These methods can be categorized into two main approaches: variational and statistical [19]. Variational methods include three-dimensional variational (3DVar) and four-dimensional variational (4DVar) approaches. The 3DVar method assimilates observations by minimizing a cost function that balances the model background and observations in a three-dimensional space at a single time point. However, it does not explicitly account for temporal evolution. In contrast, 4DVar extends 3DVar by assimilating data over a time window, leading to more accurate model forecasts as it considers the evolution of the state over time [15]. Statistical methods include the Kalman filter [20], optimal interpolation, and their derivatives: ensemble Kalman filter (EnKF) and ensemble optimal interpolation (EnOI) [21]. These methods refine numerical outputs by incorporating an ensemble of model simulation states to construct the model error covariance matrix (B). Although EnKF is more robust and widely used even today, it requires significant computational power, making its implementation a challenging and restrictive task [22].

On the other hand, EnOI has become a popular choice in ocean modeling due to its efficiency and feasibility [23–25]. Unlike EnKF or 4DVar, EnOI offers comparatively lower computational costs while still providing robust improvements to model initial conditions. This makes it particularly advantageous for forecasting systems where computational resources and time are often limited. EnOI uses a pre-established ensemble to estimate the B matrix, enabling efficient corrections and eliminating the need to update ensemble members during each assimilation cycle [26,27]. In the EnOI framework, the B matrix is defined in advance as $B\;=\;\alpha \frac{A{\;A}^{T}}{N\;-\;\text{1}}$, where A is the matrix of model anomalies sampled from the historical ensemble, N is the number of ensemble members, and α is a scalar parameter used to scale the variance to realistic levels.

Several assimilation systems have used EnOI as an assimilation scheme [28–30], with REMO Ocean Data Assimilation System (RODAS) emerging as one of the pioneers in the South Atlantic [31–34]. RODAS is the data assimilation component of the Oceanographic Modeling and Observation Network (REMO), a Brazilian initiative designed to support regional ocean forecasting and climate research.

The system has been integrated with the HYbrid Coordinate Ocean Model (HYCOM) until now, and HYCOM+RODAS is capable of assimilating gridded SST data, conventional satellite altimetric data, temperature (T) and salinity (S) profiles, and has recently conducted preliminary tests with synthetic data intended to simulate those provided by Surface Water Ocean Topography (SWOT). Due to its multivariate nature, it has been used for a wide range of applications [35–37].

Given the high computational costs of data assimilation systems employed in operational forecasting, and considering that ROMS is a widely accessible model while RODAS is a low-cost assimilation system developed for HYCOM, the objective of this study is to establish the foundation for the development of a cost-effective and optimized data assimilation framework for the ROMS model. Therefore, this work represents the first step towards the development of the new ROMS+RODAS system, which in the future will be publicly available and capable of assimilating a wide range of datasets that already integrates into the system.

## 2. Data and methods

### 2.1 Model configuration

The ROMS grid used in this study covers a significant portion of the Brazilian coast, spanning from 50.5°W to 32.5°W and 30°S to 12°S. It consists of 217 longitude points and 222 latitude points, with an approximate horizontal resolution of 0.08° and 32 vertical levels. A visual representation of this exact model domain can be found in De Paula et al. [18]. The specific areas mentioned later in this study, such as the Abrolhos Archipelago (∼18°S) and the Cabo Frio upwelling zone (∼23°S), are fully encompassed within these domain boundaries. The grid was nested within a CMEMS Analysis and Forecast run with 0.08° resolution and with 50 vertical levels. Although CMEMS Analysis currently only archives forecasts from October 2020 [38] onward on its public portal, our local archive retains the full 2008–2020 record. Initial conditions were also obtained from the CMEMS Analysis daily means. Although nested regional models are typically designed to increase horizontal resolution, the same 0.08° resolution was maintained in this study. The primary objective here was to implement and evaluate the new ROMS+RODAS data assimilation framework in a controlled regional domain. The 0.08° CMEMS data provided mesoscale-permitting lateral boundary conditions, allowing us to isolate the specific impacts of our cost-effective scheme without the prohibitive computational costs of a larger or higher-resolution domain.

Bathymetry was derived from the “1 arc-minute global relief model” ETOPO1, with a resolution of 1 minute, and corrected using data provided by the Brazilian Navy Hydrography Center (CHM). To mitigate significant variations in the principal deep-ocean water masses, a nudging of 1080 days was applied to temperature and salinity (T and S) above 400 m, and 30 days below 800 m. The nudge was computed with 10-year averages (2007–2016) from the global Mercator analysis.

The model was forced with hourly fields of temperature, relative humidity, shortwave and longwave radiation, precipitation, and wind velocity. These forcing fields were obtained from the European Centre for Medium-Range Weather Forecasts (ECMWF) Reanalysis v5 (ERA5) [39], with a horizontal resolution of 0.3°. To ensure accurate integration reproducibility, the ROMS configuration parameters were meticulously replicated following De Paula et al. [18] without any modifications.

A short spin-up was performed from October 1, 2008, to December 31, 2008. This phase was conducted to stabilize and warm up the model, and its outputs were subsequently discarded. The restart from January 1, 2009, was used as the initial condition for two experiments that ran for four consecutive years, ending on December 31, 2012: one without assimilation, named Free, and another with SST assimilation, named RODAS_SST.

### 2.2 Observational data

The RODAS_SST assimilated data from the Operational Sea Surface Temperature and Ice Analysis (OSTIA) provided by the UK MetOffice, available at https://data.marine.copernicus.eu/product/SST_GLO_SST_L4_NRT_OBSERVATIONS_010_001/services/. OSTIA offers daily SST maps with high spatial resolution (0.05° x 0.05°), generated from data collected by satellites, ship-based stations, moored buoys, and drifting buoys. These data are produced by the Group for High Resolution Sea Surface Temperature (GHRSST) [40,41]. The L4 version of the data was used due to its extensive processing, rigorous quality control, and robust gap filling. The OSTIA fields were interpolated onto the study grid and used only in the assimilation experiment, being assimilated exclusively at grid points fully surrounded by water and with depths greater than 100 meters. This choice was justified due to the high errors of OSTIA in shallow and coastal areas [40].

### 2.3 REMO ocean data assimilation system

Regarding the assimilation system, RODAS essentially operates in two versions that differ primarily in how the innovation is computed in the analysis equations. The first and older version follows the methodology proposed by Thacker and Esenkov [42] and was later used by Dorfschäfer et al. [33] and Mignac et al. [35]. This procedure involves creating and assimilating a synthetic isopycnal layer thickness derived from the observed T/S profiles. The data are interpolated, and the innovation is computed directly within the model’s isopycnal layers. The second was introduced to RODAS by Costa and Tanajura [34]. It allows the innovation to be computed in the vertical space of the observations. This study opted to use the latter approach, as the authors demonstrated through objective comparisons that this method provides superior results compared to innovations computed in isopycnal layers.

One of the challenges of ensemble methods is determining the sufficient number of members to adequately capture the model state without increasing computational cost. The EnOI uses a set of model anomalies from a free run to estimate the **B** matrix, which directly influences the computation of the objective analysis. In this study, the ensemble consisted of 90 members derived from a six-year integration, from January 1, 2009, to December 31, 2014. In this study, the assimilation frequency was set to every three days. For each cycle, the ensemble was constructed using a 45-day window centered on the analysis day, sampling 15 members per year at three-day intervals. By applying this window over a six-year historical integration (January 1, 2009, to December 31, 2014), the procedure resulted in a total of 90 members. Previous works such as Tanajura et al. [32] and Santana et al. [37] employed 126 members, however, in this study we opted for 90 members in order to keep the computational cost manageable while still ensuring robust representativeness, as verified through prior sensitivity tests. The 45-day window assumes that the background errors are equivalent to the model’s small-scale temporal variabilities and is commonly applied in studies involving EnOI [26,29,43].

The equations governing the analysis are presented in Eq. (1) and Eq. (2), with the latter representing the computation of the Kalman gain matrix (K) [28,44].


$$\begin{array}{c} w^{a}\;=\;w^{f}+\;K\left(\text{y}-\;Hw^{f}\right) \end{array}$$ (1)

$$\begin{array}{c} K=(C\circ B)H^{T}{\left[H(C\circ B)H^{T}+R\right]}^{-\text{1}} \end{array}$$ (2)

in the analysis equation (Eq. 1), w  represents the model state vector, where the superscripts a  and f  denote the analysis and forecast, respectively. y is the observation vector and the operator H interpolates the model fields onto the observation space, ensuring comparability between model and observed values. The term $(y-\;Hw^{f}\backslash )$ defines the innovation vector, i.e., the mismatch between observations and the model background. The Kalman gain matrix (Eq. 2) weights this innovation to update the state, minimizing the error of the analysis with respect to observations. The B matrix characterizes model uncertainties, while the observation error covariance matrix (R) accounts for observational errors. When R is small (reliable observations), more weight is given to the observations, whereas a small B (reliable model background) favors the model. For this study, the observational errors populating the diagonal of the R matrix were extracted directly from the spatially and temporally variable “analysis_error” field provided within the OSTIA NetCDF files.

A localization operator (C) is applied to suppress spurious long-range correlations in B, following Gaspari and Cohn [45]. Here, both C and the radius of influence are explicitly defined as 100 km, guaranteeing that all interior grid points contribute to the analysis while spurious distant correlations are minimized. It is important to note that the localization matrix C is strictly two-dimensional (horizontal). To prevent spurious covariances from inappropriately updating deep ocean layers based solely on surface observations, no explicit vertical localization function was applied; instead, the assimilation increments were strictly restricted to the upper 17 vertical layers of the model (approximately down to 300 m depth). The final analysis increment, represented by the right-hand side of Eq. 1, reflects the correction applied to the model state after assimilation.

In our EnOI implementation, the ensemble is used to estimate the B matrix, which is then scaled by a weighting factor α = 0.5 when computing K [21]. This α attenuates the climatological spread to realistic levels but does not constitute a multiplicative inflation as in EnKF. Unlike EnKF, which employs inflation factors greater than one to counteract dispersion in a dynamically evolving ensemble, the EnOI weighting α merely adjusts our confidence in the fixed covariance estimate without evolving the ensemble over time.

### 2.4 Oceanic variables

The oceanic variables analyzed include SST, sea surface height (SSH), velocity components (u, v), and temperature and salinity fields throughout the water column. These variables are fundamental for evaluating the large-scale ocean circulation, thermohaline structure, and energy exchanges within the model domain. Besides the variables that make part of the model state vector, other variables were also analyzed. The quantification of the transport of the Brazil Current (BC) and its variability is essential for understanding regional ocean circulation and its climate impacts.

Previous studies have provided detailed estimates of the variability of the BC transport on interannual and seasonal scales, based on observations and reanalyses [46–49]. In this work, the BC transport was estimated from sections performed at various latitudes, ranging from 18°S to 28°S. The objective was to evaluate the impact of SST assimilation on the magnitude and variability of the BC transport in comparison with the Free run. Mean fields from the four years of simulation were computed, and only small differences were observed, likely due to the relaxation of T and S applied in both experiments. This additional term modifies the thermohaline gradients and, consequently, the density of the water column, which in turn affect currents intensity and transport estimates. Therefore the mean transport was then calculated for the first three months of integration, when the increments tended to be more relevant. The calculation was performed at each section based on the distance, in kilometers, between pairs of grid points (lat/lon) from the surface down to 400 m. This range was chosen based on Schmid & Majumder [47], who indicated that, north of 27°S, this depth reasonably represents the extent of the BC. The distance between grid points was calculated using standard spherical distance formulas.

After determining the velocity components, they were multiplied by a rotation matrix to ensure that the directions remained perpendicular to each section. Then, the volumetric transport was calculated according to:


$$\begin{array}{c} T\;=\int_{\text{0}}^{\text{4}\text{0}\text{0}}\int_{a}^{b}vdxdz \end{array}$$ (3)

where v  is the velocity component perpendicular to the section from the surface down to

400 m [47] and b−a  represents the width of the horizontal cell almost always orthogonal to the coast.

SST plays a crucial role in oceanic heat content (HC) as it constitutes the primary heat exchange interface between the ocean and the atmosphere. Ocean HC is essential for regulating the global climate, storing vast amounts of thermal atmospheric energy directly influencing the planet’s energy balance. Accurate HC monitoring is vital for initializing weather and climate forecasts, given that HC carries information capable of impacting phenomena such as the El Niño Southern Oscillation (ENSO) [50] and the Atlantic Meridional Overturning Circulation (AMOC) [51]. Meinen and McPhaden [52] and McPhaden [53] describe how oceanic HC influences SST anomalies in the equatorial Pacific during El Niño episodes.

Inaccuracies in HC estimation can lead to significant consequences, such as inadequate predictions of global temperature changes and underestimating or overestimating the intensity of hurricanes and tropical cyclones [1], directly affecting anthropogenic responses to natural disasters. Furthermore, these inaccuracies may impact sea level predictability due to thermal expansion and influence marine biodiversity, as temperature variations can affect species sensitive to long-term changes [54]. In this study, HC was computed using the following equation:


$$\begin{array}{c} HC\;=\;\sum i\;\rho_{i}\;c_{pi\;}T_{i}\;\Delta z_{i} \end{array}$$ (4)

here, ρ represents density, $c_{p\;}$ is the specific heat capacity, T is the temperature, Δz is the layer thickness and i is the vertical layer index.

Estimates of latent heat (LH) and sensible heat (SH) fluxes are typically obtained through parameterizations that utilize functions of surface meteorological variables, such as wind speed, humidity and temperature gradients between the air and the sea [55]. Due to the critical role that SST plays at this interface, the LH and SH fluxes were computed for both the Free run and the RODAS_SST analysis. These fluxes were calculated following Parkinson and Washington [56]:


$$\begin{array}{c} SH\;=\;\;\rho_{a}\;c_{p\;}C_{h}\;V_{wg}\left(T_{a}\;-\;T_{sfc}\right) \end{array}$$ (5)

$$\begin{array}{c} LH\;=\rho_{a}\;LC_{e}\;V_{wg}\left(q_{\text{1}\text{0}}\;-\;q_{s}\right) \end{array}$$ (6)

where $\rho_{a}$ is the air density, $c_{p}$ is the specific heat of dry air, L  is the latent heat of vaporization, $C_{h}$ and $C_{e}$ are the transfer coefficients for sensible and latent heat, respectively. $V_{wg}$ is the geostrophic wind speed, $T_{a}$ is the air temperature, $T_{sfc}$ is the SST, $q_{\text{1}\text{0}}$ is the specific humidity at 10 m, and $q_{s}$ is the surface specific humidity. All surface meteorological inputs are obtained from the ERA5 reanalysis, while $T_{sfc}$ is the prognostic SST output from ROMS.

Also, to assess the statistical significance of the differences between the Free and RODAS_SST experiments, the Wilcoxon signed-rank test was applied to the daily domain-averaged and time series. Statistical significance was defined at (p < 0.05).

## 3. Results

### 3.1 Comparison with SST observations

Fig 1 presents the OSTIA SST fields (a, b, c), the SST errors of the experiments relative to OSTIA (d–l), and the analysis increments (m, n, o) for two selected illustrative days (January 1, 2009, and July 2, 2012) as well as their overall temporal average. In all panels, the black dashed line indicates the 100 m isobath, which delineates the spatial boundary of the assimilation domain. All runs were evaluated at three-day intervals, corresponding to the assimilation cycle frequency, where observations were incorporated every 72 hours and new simulations were initialized from the updated model state.

**Fig 1:**
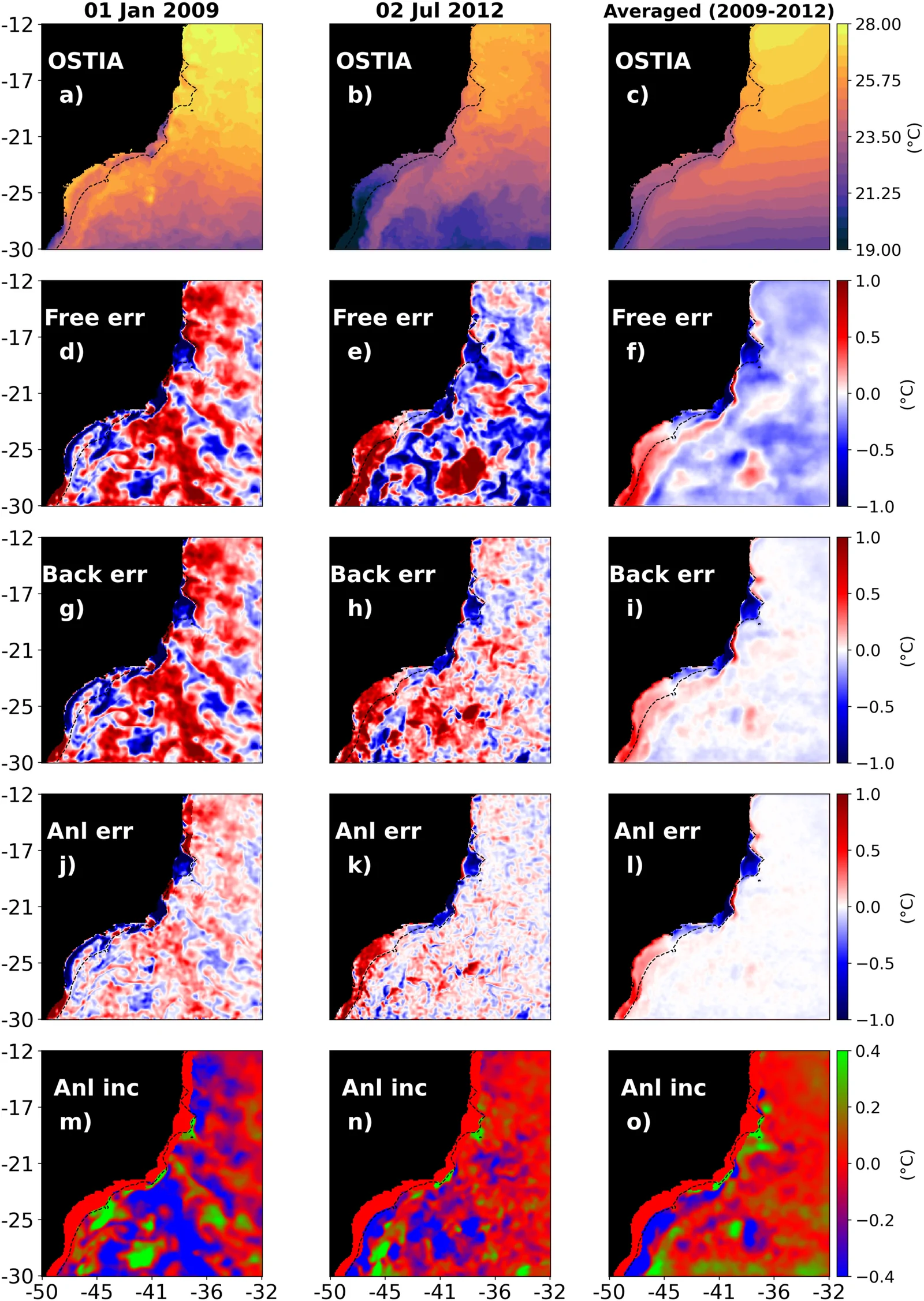
OSTIA temperature maps (a, b, c), temperature difference maps (experiments – OSTIA) (d-l), and analysis increment maps (m, n, o). The dashed black lines indicate a 100 m depth.

On the first assimilation day (January 1, 2009), both the Free and background fields exhibited errors reaching up to 1°C in shallow coastal waters and in regions dominated by the Intertropical Convergence Zone (ITCZ) and South Atlantic Convergence Zone (SACZ). These elevated initial errors are commonly linked to intrinsic forecasting system limitations [57] and complex ocean-atmosphere feedback mechanisms [58]. Specifically, intensified cloud cover in these zones induces local ocean cooling [59] and severely complicates accurate satellite SST sampling [60], leading to artificial positive anomalies in the models. However, following the assimilation, the RODAS_SST analysis (Fig 1J) demonstrated a robust correction of these biases, significantly reducing errors to below 0.5°C across most of the open ocean domain.

The analysis map of RODAS_SST (Fig 1J) showed substantially smaller errors compared to the map of Free (Fig 1D), with values below 0.5°C across most of the domain. Assimilation proved beneficial, correcting a substantial portion of the errors in the open ocean while leaving the shelf errors unchanged. The largest SST errors were also found in regions associated with the previously discussed meteorological phenomena, where errors were slightly higher but never exceeded 1°C, as shown in Fig 1D and 1G.

The second column of Fig 1 displays the same variables as the first column but for the final year of the integration period (July 2, 2012, which corresponds to winter in the Southern Hemisphere). It is evident that the errors presented by the Free experiment (Fig 1E) remain as high as those from Fig 1D. However, unlike in Fig 1D, ROMS underestimated SST relative to OSTIA, except in two regions: one on the southern shelf of the domain and another between 25°S and 30°S and 41°W to 36°W. The errors of the background field (Fig 1H) were substantially smaller than those shown by the Free run (Fig 1E) and also smaller than the errors in Fig 1G, where no assimilation had yet been performed. The analysis field (Fig 1K) displayed errors that were mostly smaller than those of the background fields (Fig 1H) and also smaller than its own fields from earlier dates, when fewer assimilation cycles had been implemented (Fig 1J).

When observing the mean error of each run (last column), it is evident that ROMS exhibits a predominantly negative bias, with SST values frequently lower than those presented by OSTIA across almost the entire domain (Fig 1F). These differences were close to −0.5°C, except in areas near the shelf and in a dipole zone between 25°S and 30°S, where a positive bias is observed. The regions with the largest errors are associated with coastal areas, such as the Abrolhos Archipelago (~18°S) and the highly variable Cabo Frio upwelling zones (~22°S). The background fields mitigated this bias to values below 0.25°C (Fig 1I), indicating that the 72-hour forecasts retain a good memory of the assimilated fields, remaining closer to the OSTIA fields than the Free run. The average map of the analysis error (Fig 1L) effectively reduced the bias from the Free run almost entirely, presenting values consistently below 0.1°C, except in regions with depths shallower than 100 m.

The last row of Fig 1 shows the SST values added by RODAS in the background field, known as the analysis increment. It is noted that the most intense analysis increments (Fig 1M–1O) were always opposite in sign to the largest errors shown by the 72-hour forecast (Fig 1G–1I). This behavior is mathematically expected because the 72-hour forecast serves as the background state for the assimilation cycle; hence, the increments are computed precisely to counteract its deviations from the observations.

A negative increment indicates cooling induced by the assimilation. Authors have shown that the presence of the SACZ tends to reduce ocean SST immediately below it, leading to errors in observational sampling [61]. Pezzi et al. [62] concluded that the convergence of atmospheric fluxes combined with changes in Ekman pumping in the presence of clouds in the southwestern Atlantic, causes sea surface cooling through dynamic and thermodynamic processes. The Free run was unable to simulate these effects, leading to the necessity of negative increments through assimilation.

On the other hand, a positive increment indicates the necessity to increase the SST values in the background fields to bring the objective analysis closer to the observations. For example, the positive increments along the contours in Fig 1O aimed to mitigate the negative bias present in the contours of Fig 1I. The sign of the increments in the dipole at 26°S was also reversed to attenuate this phenomenon, which was produced by the Free run and is absent in the observations. The presence of these higher errors in the contours of Fig 1I suggests that the model’s integration dynamics may contribute to the persistence of these errors, as this pattern is hardly visible in the analysis average error map (Fig 1L).

The SST corrections demonstrated in Fig 1 were achieved using a 3-day assimilation window. Studies on ocean DA have shown that the frequency of assimilation cycles can significantly impact these results. For instance, Sandery [63] compared 1-day assimilation cycles with 3-day cycles, finding that more frequent cycles improve forecast accuracy for variables including SST. While 1-day cycles could have further pronounced the error reductions seen in Fig 1, this would have tripled our computational cost, contradicting the main operational advantage of the EnOI scheme in RODAS. To quantitatively illustrate this cost-effectiveness based on our hardware setup, a single EnOI cycle takes less than 30 minutes. Following Kerry et al. [43], who estimated 4DVar to be roughly 25 times more expensive than EnOI, a single 4DVar cycle would require about 12.5 hours. Furthermore, an EnKF implementation employing 90 ensemble members – scaling the cost by N = 90 – would require approximately 45 hours per cycle. Extrapolating these costs to the 487 cycles performed during our 4-year integration, the EnOI scheme completed the assimilation steps in roughly 10 days of continuous computation. In contrast, equivalent integrations would take approximately 8.5 months using 4DVar and over 2.5 years using EnKF. This contrast has the potential to justify the operational feasibility of ROMS+RODAS for research groups with constrained resources.

Fig 2 shows the root mean square deviation (RMSD) relative to OSTIA data calculated for the 487 days during which assimilation was performed. Since the application of the EnOI scheme within the RODAS framework for this specific regional ROMS configuration is a novel implementation, evaluating the RMSD against the assimilated dataset (OSTIA) is a mandatory first step. This dependent validation serves as a crucial internal consistency check to mathematically prove that the assimilation system is functioning correctly and effectively pulling the model state toward the observational reference. It is important to reiterate that the assimilation system does not assume these observations are perfect, as their uncertainties are weighted by the variable observation error matrix (R) derived from the OSTIA products.

**Fig 2:**
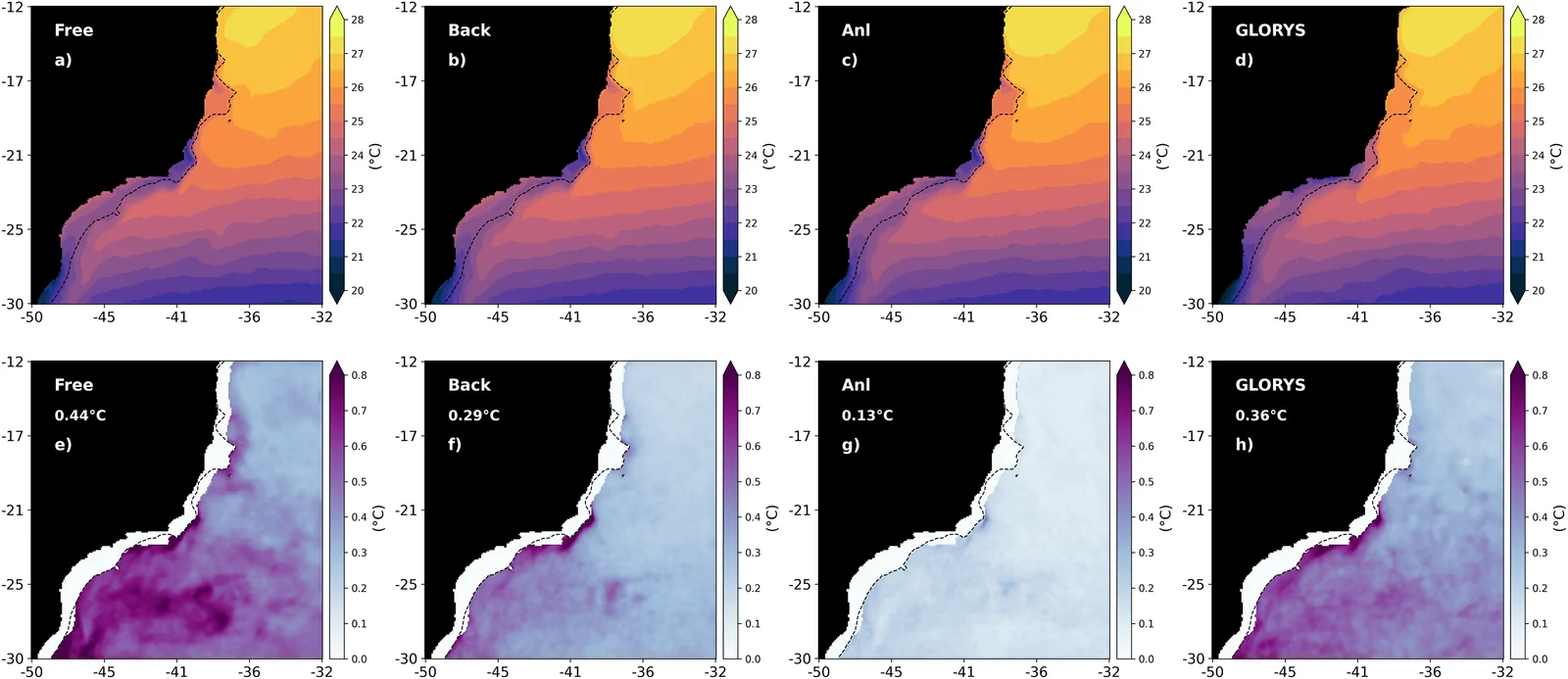
Mean temperature for the 4 years (2009–2012) of ROMS integration (a-d) and Root Mean Square Deviation (°C) using 487 numerical outputs relative to OSTIA fields (e-h).

For benchmark purposes, fields from the Global Ocean Reanalysis from the CMEMS (GLORYS), with 1/12° horizontal resolution, were interpolated onto the ROMS grid and used in the comparison. The Free run exhibited the highest RMSD values, exceeding 0.8°C in several regions, particularly in the southwestern section of the study area, areas near the slope influenced by the BC, and in the open ocean where the SACZ strongly impacts south of 25°S. When compared to the background field, a significant reduction in the mean RMSD is observed, from 0.44°C to 0.29°C, corresponding to only 65% of the value obtained in the run without assimilation. This reduction confirms the results in Fig 1, demonstrating the benefit of data assimilation in improving the quality of short-term forecasts. This finding aligns with Vargas et al. [64], where the authors assimilated lightning data into the Weather Research and Forecasting model and observed improvements in the system’s high-frequency outputs. It is important to note that the RMSD calculation was performed only for the days when assimilation took place.

The RODAS_SST analysis map shows the lowest RMSD values among all those presented, with a spatial average of 0.13°C. These errors rarely exceeded 0.3°C, except in the zone near the 100 m isobath, close to Rio de Janeiro (21.5°S, 29°W). RODAS was able to reduce the RMSD values of the Free run by 70%, the 72-hour forecasts by 55%, and GLORYS (0.36°C) by 64%. However, it is important to note that GLORYS does not directly assimilate OSTIA data but rather data from the Advanced Very High Resolution Radiometer (AVHRR), which captures a portion of the aggregated OSTIA data. The AVHRR measures radiation across different bands of the electromagnetic spectrum, particularly in the infrared, allowing for SST detection even at night. Therefore, a fairer comparison would be between the background field and GLORYS, since, as mentioned earlier, the RMSDs of the 72-hour forecasts were always computed using non-assimilated data. The direct evaluation of the RODAS_SST analysis fields against the assimilated OSTIA data was maintained primarily as a proof of concept. Because this study represents the first implementation of RODAS into ROMS, evaluating the analysis against the ingested observations was a necessary step to confirm that the newly coupled assimilation scheme successfully pulls the model state toward the observations. This verifies the structural correctness of the system, ensuring that any indirect degradations (e.g., in subsurface layers) are strictly due to non-ideal parameter configurations – such as spurious covariances – rather than fundamental coding or implementation errors.

The background presented an average RMSD approximately 20% lower than the RMSD from GLORYS, correcting a significant portion of the southern domain and the continental slope, region where the western boundary currents flow. Only RODAS_SST managed to eliminate the high RMSD values, around 0.7°C, in Cabo Frio (~22°S, near the 100 m isobath). These high values may be generated due to the wind pattern and the abrupt change in coastline direction in this region. Fox-Kemper et al. [65] comprehensively discussed the difficulties faced by ocean circulation models in addressing various challenges, including cases with complex bathymetry and intense temperature gradients. They emphasized the need for higher resolutions and more adequate parameterizations to improve the accuracy of numerical forecasts. In this work, we managed to improve the representation of this region but further improvements are still necessary.

To investigate the evolution of the quality of SST analysis increments produced by RODAS, Fig 3 presents the time series of the daily RMSD from 487 numerical simulations relative to OSTIA data. A Gaussian filter was applied to facilitate visualization of the curves. Errors from all simulations exhibit a marked seasonal variation, with higher values during the summer months in the Southern Hemisphere. This variability may be influenced by external seasonal factors, such as solar radiation cycles, changes in heat transport, precipitation, wind patterns, and other natural processes that vary throughout the year. In a recent publication, Mayer et al. [66] reported that in the Indian Ocean region, sources of errors in forecasts can be directly associated with the spatiotemporal distribution of the thermocline, where shallower thermoclines showed greater errors. Additionally, the authors attributed these errors to the initial conditions used.

**Fig 3:**
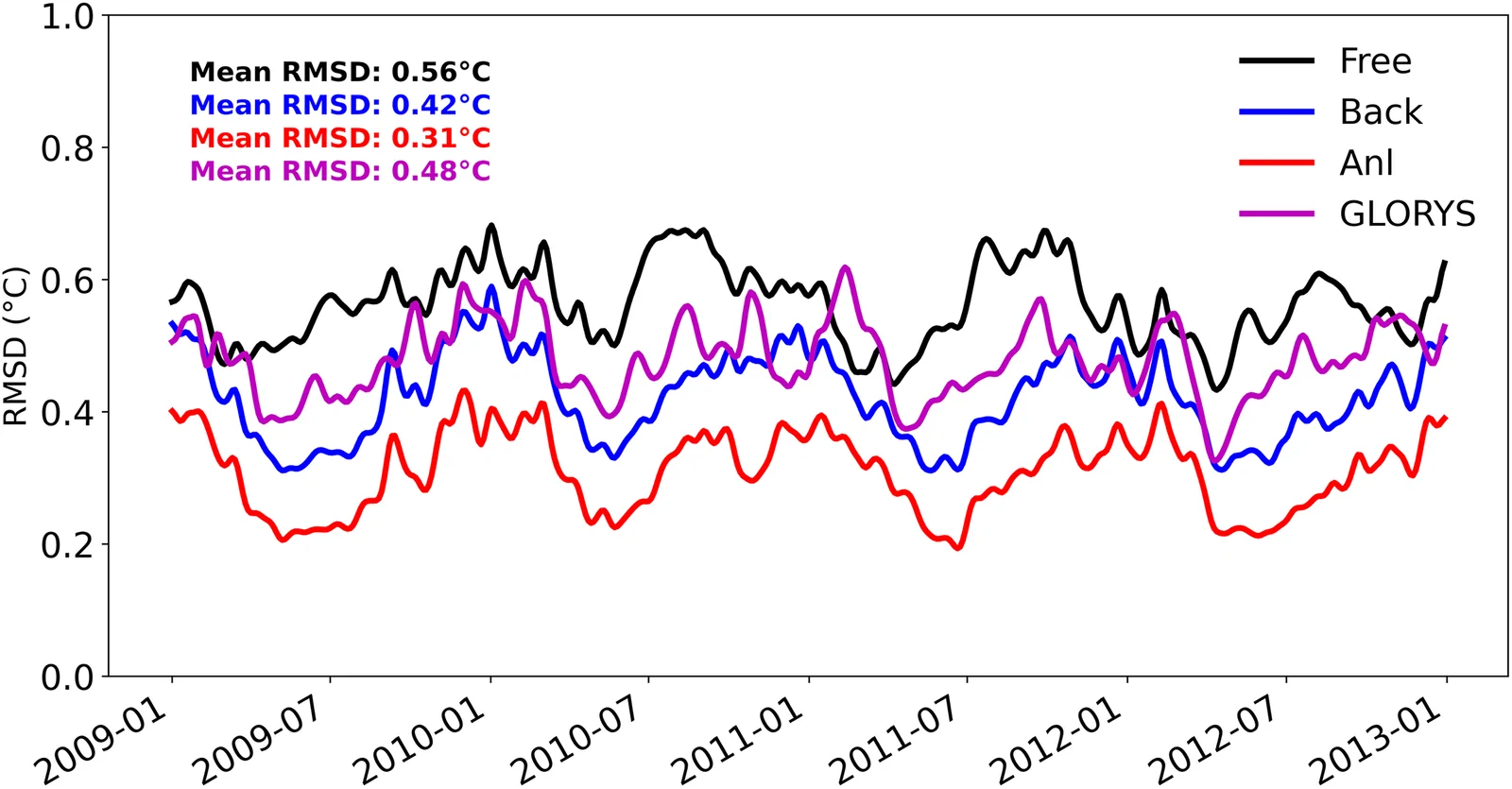
Time series of the daily average Root Mean Square Deviation (°C) for the 487 fields from the Free run (black), background (blue), objective analysis (red), and GLORYS (purple) relative to OSTIA data, covering the period from January 1, 2009 to December 31, 2012.

A closer inspection of Fig 3 also reveals a slight temporal lag in the seasonal variability of the RMSD between the Free run and the assimilated runs (background and analysis). This phase shift can be attributed to two main interconnected factors. First, the continuous SST updates modify the upper ocean’s thermal stratification and heat content (as discussed later in Section 3.4), thereby altering its thermal inertia. This causes the assimilated ocean state to respond to seasonal atmospheric forcings at a slightly different rate compared to the unconstrained Free run. Second, the EnOI scheme utilizes a 45-day moving window to sample the historical ensemble and compute the background error covariances. This temporal averaging inherently carries a memory of recent past states, which may introduce a slight delay in the assimilation system’s adjustment during rapid seasonal transitions.

The Free run showed the poorest results, with an average RMSD of 0.56°C. The background curve exhibited an average RMSD of 0.42°C, indicating a 25% improvement compared to the free simulation, suggesting that the increments added three days earlier persist partially over the integration time. This curve was intermediate between the Free curve and the analysis curve. The latter again presented the lowest average RMSD of 0.31°C, representing a 45% improvement over the Free run, a 26% improvement over the 72-hour forecast, and a 35% improvement compared to the RMSD of GLORYS (0.48°C). GLORYS showed intermediate performance, with an RMSD higher than the model’s analysis but lower than the Free simulation. Although GLORYS is a high-quality reanalysis, using different data or alternative assimilation methods can lead to differences in SST products.

The analysis consistently shows improvement throughout the entire sampled period. Notably, the analysis curve is the only one that does not intersect with the others, highlighting the consistent enhancement incorporated into the background field when all simulations are compared to OSTIA.

To quantitatively confirm the observed improvements, the non-parametric Wilcoxon signed-rank test was applied to the RMSD distributions. The reduction in the daily SST RMSD time series achieved by the RODAS_SST analysis relative to the Free run was highly statistically significant (p < 0.001). Furthermore, the pixel-by-pixel spatial evaluation of the mean RMSD maps corroborated this consistent error reduction across the oceanic domain, also yielding p < 0.001. These statistical results confirm that the SST assimilation effectively pulled the model state toward the observational reference. Thus, Figs 2 and 3 demonstrate the positive impact of RODAS on SST simulation, which could significantly benefit predictions of directly dependent variables such as HC, latent heat flux, and sensible heat flux. These advancements have the potential to enhance climate forecasts, where SST is a key oceanic component [67] and plays a crucial role as a boundary condition for coupled models [68].

### 3.2 Three-dimensional performance assessment

Evaluating the thermohaline performance in subsurface, Fig 4 presents the vertical RMSD profiles calculated against independent Argo (Array for Real-time Geostrophic Oceanography) profiles [69,70] within the domain, assessed on the analyses days. Initially, 441 profiles were identified, but after removing spurious profiles, 420 valid profiles remained. The simulation errors were low, with RMSD values not exceeding 1.6°C for temperature and 0.4 PSU for salinity. Analyzing the Observing System Experiment (OSE) that assimilated SST and SLA data but excluded Argo profiles, Oke et al. [71] using the Mercator System, reported maximum RMSD values of approximately 1.1°C for temperature and 0.3 PSU for salinity. These results highlight that despite the lack of Argo assimilation, the system’s performance remained robust, yielding errors consistent with other studies in the literature, such as [32].

**Fig 4:**
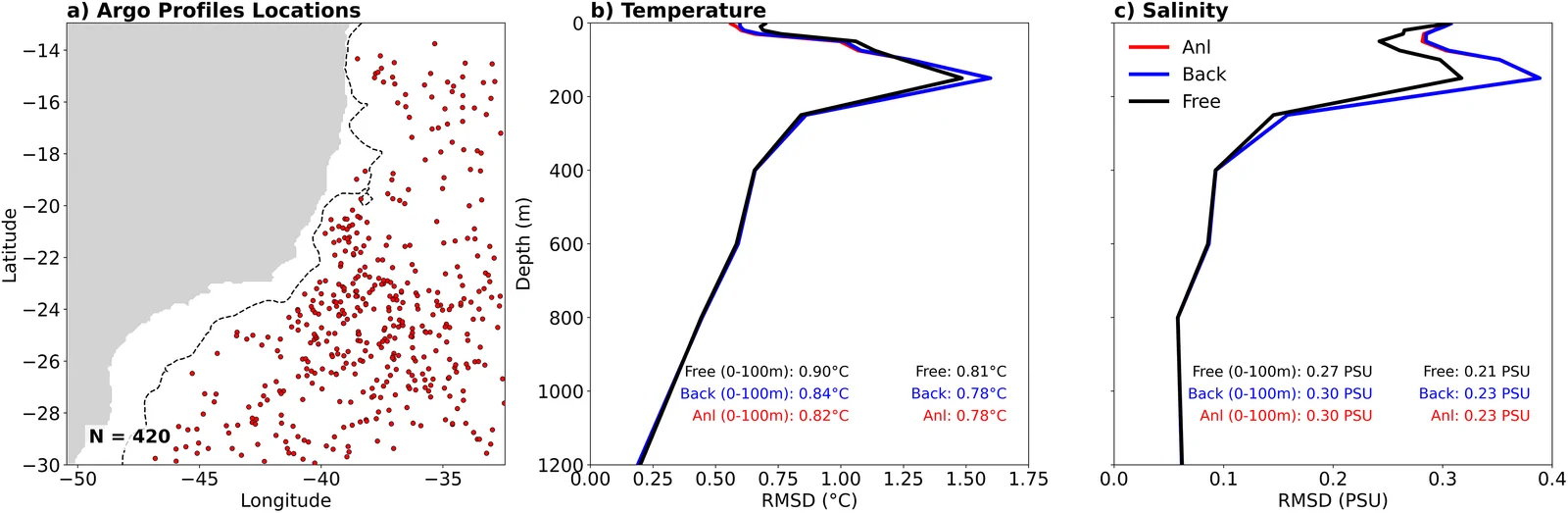
Validation of the model experiments against Argo profiles. (a) Spatial distribution of the 420 Argos (N) used for the assessment, with the black dashed line representing the 300 m isobath. (b) Mean vertical profiles of Root Mean Square Difference (RMSD) for temperature (°C) and (c) salinity (PSU) from the surface down to 1200 m depth. The lines denote the Free run (black), Background (blue), and Analysis (red). Domain-averaged RMSD values for the entire water column and specifically for the upper 100 m are explicitly shown within the respective panels.

For temperature, there were a few relevant differences between the simulated fields. Within the first 100 m of the water column, the Free run exhibited the highest RMSD values, with an average curve of 0.90°C. Assimilation of SST reduced this value to 0.82°C, representing a decrease of approximately 10%, with the largest discrepancies observed in the upper ocean layers. The difference between the background mean (0.84°C) and the analysis was small, but it can be noted that the analysis increments for temperature were predominantly positive down to subsurface depths. Between 100 and 250 m, a region of thermocline variability, there was a degradation in the temperature of the analysis curve compared to the Free run because variations in SST do not correlate well with temperature changes at these depths. Models often face challenges in the accurate simulation of thermocline and pycnocline regions, where vertical gradients are pronounced [25]. Miyazawa et al. [72] assimilated SST data from the Moderate Resolution Imaging Spectroradiometer (MODIS) and also observed degradations in T and S fields, especially in the upper ocean (up to 400 m). The authors attributed these errors to the noise from the SST sensor, which introduced inconsistencies into the fields, and suggested better calibration of the sensor and its derived products. Tanajura et al. [31] proposed that the degradation of subsurface salinity could be linked to inaccurate covariances introduced by the ensemble members in the B matrix. In this study, we used the Free run as the ensemble, and it was found that the largest errors, for both T and S, followed the pattern observed in the Free run. Kerry et al. [43] suggested that such degradation is inherent to the EnOI scheme.

For salinity, the increments generated by the EnOI covariances tended to increase the errors from the Free run throughout most of the upper ocean (up to 400 m). Kerry et al. [43] compared the salinity of assimilation systems with independent glider data and also found larger errors using the EnOI below 200 m, with a difference of 0.07 PSU between the EnOI and 4D-Var runs. Although the average difference found, 0.02 PSU, is not statistically significant, possible field degradation cannot be ruled out.

The Wilcoxon signed-rank test was also applied here to the vertical RMSD profiles computed against the Argo dataset. For temperature, the RODAS_SST experiment showed a statistically significant error reduction in the upper 100 m (p < 0.001) compared to the Free run. However, when evaluating the entire water column (0–2000 m), the temperature differences between the runs were not statistically significant (p = 0.249). This reflects the surface-restricted nature of the assimilation, which successfully constrains the upper thermal structure without imposing spurious, large-scale artificial changes in the deep ocean. Conversely, for salinity, the test confirmed that the subsurface degradation introduced by the EnOI cross-covariances is statistically significant (p < 0.001). These metrics corroborate that while surface-only assimilation effectively improves the upper-ocean thermal state, it highlights the need for vertical localization or independent salinity profile assimilation to prevent physically inconsistent multivariate increments in deeper layers.

The use of the Free run as the ensemble in the RODAS_SST experiment appears to have contributed to the increase in errors, as this run did not incorporate thermohaline corrections from the assimilation of Argo profiles [71]. Studies by Costa and Tanajura [34] and Santana et al. [37] also observed subsurface salinity degradation when only T data were assimilated using the EnOI. We are still investigating the responses of ROMS to the updating of variables through assimilation, as well as determining the optimal configuration for the new system under development. This work aims to identify and mitigate the observed issues and enhance the system’s performance with a data assimilation framework.

Still investigating T using objective metrics, Fig 5 compares the Free run and the RODAS_SST analyses with independent data from the PIRATA buoy located at 19°S and 34°W. An effort was also made to compare salinity against the PIRATA buoys. Although the observational time series suffered from extensive gaps, the available data indicated a slight degradation in the SSS fields following the SST assimilation. We opted not to present these highly fragmented plots, as this localized negative impact on salinity is entirely consistent with the broader 3D subsurface degradation already extensively discussed in the Argo validation section. In the first 100 m (Fig 5D and 5E), it is possible to observe a bias of lower temperatures in the Free run compared to the observations. The errors frequently reached values close to −1°C. Data assimilation proved beneficial as the negative structures were mitigated both in dimension and intensity. In Fig 5E, it is noticeable that the errors in this part of the ocean are represented with lighter and more spaced colors, indicating less intensity. Assimilation by RODAS corrected a significant part of the T bias present from the surface to a substantial portion of the upper euphotic zone. This is the most superficial part of the photic zone, where sunlight is most intense, enabling photosynthesis to occur efficiently. It is in this zone that most photosynthetic organisms, such as algae and phytoplankton, thrive, supporting a rich marine biodiversity [73]. The most intense corrections for this oceanic layer can be observed starting in April 2012 near the end of the series.

**Fig 5:**
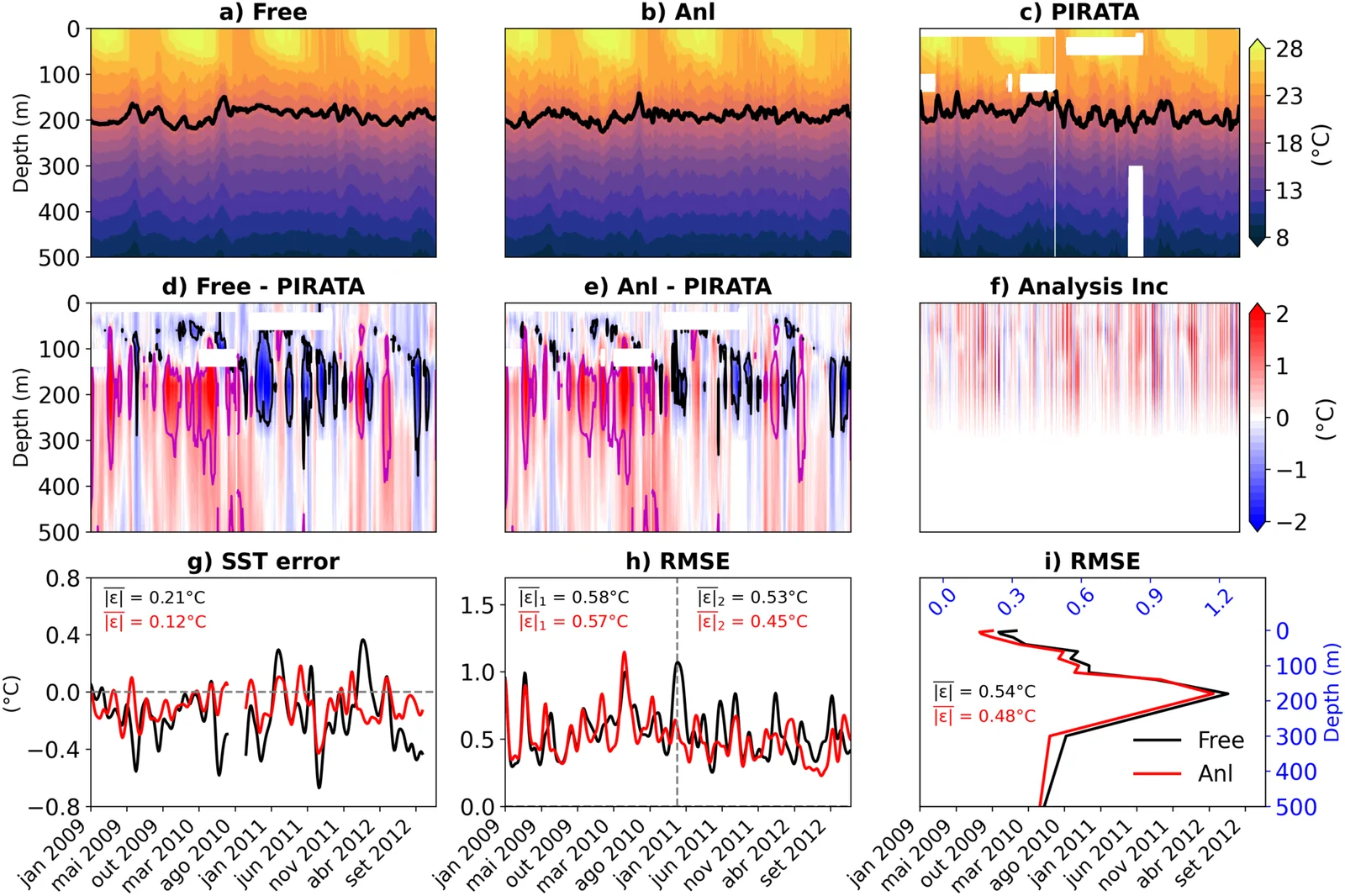
Time series of temperature and the 20°C isotherm depth (a, b, c), temperature error from the experiments (d, e), and analysis increments (f). Time series of SST error (g), time series of RMSD for the entire water column (h), and vertical RMSD profile by depth level (i). The experiment data were all vertically and horizontally interpolated to the PIRATA buoy located at 19°S, 34°W. |ɛ| represents the mean absolute error of the curves.

Between 100 and 300 m, several corrections are evident. For instance, structures with errors equal to or greater than 1.5°C between 2009 and 2010 were attenuated to values close to 0.5°C in the analysis error graph due to assimilation. Furthermore, the large range with predominantly negative bias was significantly reduced, particularly between August 2010 and January 2011, where a large portion of the water column in the Free run showed errors close to −2°C. An exception is the interval between 150 and 250 m, starting from September 2012 and at the end of the series, where there was an increase in error despite corrections in the above layers. This result is corroborated by observing the RMSD graph in Fig 5H, and its probable mechanisms were discussed in the analysis of Fig 4.

Fig 5G shows that part of the largest correction amplitudes at the surface are located precisely at the end of the series, starting in April 2012. A possible explanation for this inconsistency may lie in how the increments in Fig 5F were applied, degrading part of the subsurface. In EnOI, the B matrix is directly estimated through the variability of the model itself and it does not evolve dynamically over time. This simplifies and accelerates the assimilation process but may limit the accuracy of results by failing to capture real variability in the system’s most recent conditions. As a result, assimilation may not accurately reflect the variables in some specific regions, such as the thermocline zone or deeper ocean areas. This loss of quality was discussed earlier and typically occurs due to the spurious covariances between the simulated variables during the calculation of the B matrix. To prevent unrealistic increments from being introduced via covariances, some authors, such as Santana et al. [37], applied vertical localization in the assimilation of T/S profile data. According to Wang et al. [74] the introduction of vertical localization in SST assimilation using isopycnal coordinates improves system performance throughout the water column, reducing degradation at intermediate depths (2000–4000 m) and increasing the overall accuracy of the analysis, especially in the North Pacific and North Atlantic. The present study did not find degradation at such great depths since we opted to apply increments only up to the maximum depth corresponding to the seventeenth layer of ROMS. However, if vertical localization was applied, the gains resulting from covariances with greater depths could possibly be mitigated or even nullified. This aspect may be considered in future works, as we are still working on the development and adjustment of the system. Besides, in a recent publication, Stanley et al. [75] showed that the Gaspari-Cohn method may not be a good choice for cross-fluid vertical localization in an atmosphere-ocean coupled system. A plausible solution might be to apply vertical localization of SST up to the mixed layer depth (MLD), subsequently allowing T/S profiles to correct the deeper parts of the system.

Although there were no increments below 300 m at the PIRATA point, contrary to the discussion in Fig 4, we observed that most corrections were predominantly positive. The improvements propagated downward through the model’s integration dynamics, refining the accuracy of deeper layers over time. This occurs mainly via internal waves, advection, and vertical diffusion, which transport the influences of corrections made in upper levels to deeper layers [76].

Fig 5G shows that the objective analysis outperformed the Free run during almost the entire study period, reducing the mean SST error of the ROMS without assimilation from 0.21°C to 0.12°C, an approximate reduction of 43%. It is also evident that the model’s negative bias was attenuated by the assimilation, with some points showing significant corrections close to 0.5°C. To properly quantify the visual reduction of subsurface errors observed between panels d) and e) of Fig 5, we can examine the RMSD metrics explicitly detailed in panels h) and i). Fig 5H presents the daily RMSDs for the entire water column. Analyzing the graph, we note that unlike Fig 5G, where the assimilation gains were significant from the start of the integrations, the corrections imposed along the water column were much more notable in the second half of the integration period, where the implementation of RODAS managed to reduce the RMSD by approximately 15%, from 0.53°C to 0.45°C. As mentioned earlier, this occurs because the corrections take time to reach greater depths, being transferred downward mainly through turbulent diffusion and vertical convection schemes [9].

Finally, Fig 5I shows the average RMSDs for each depth level at the PIRATA buoy. The data indicate that the assimilation run performed better at all depth levels up to 500 m, even with increments limited to depths shallower than 300 m. The RODAS_SST analysis showed an average RMSD of 0.48°C, a 11% reduction compared to the Free run RMSD (0.54°C). This result suggests that SST assimilation can benefit deeper layers even when it occurs only every three days. The reason for the difference when comparing with Argo dataset, is not yet fully understood and could be a topic for future discussions.

The correlations between the runs and the absolute dynamic topography (ADT) from the Archiving, Validation and Interpretation of Satellite Oceanographic data (AVISO) showed little difference between the Free and RODAS_SST runs (graphs not shown). Calculating the correlations over the 487 analysis days, the Free experiment showed an average correlation of 0.54, while RODAS_SST showed 0.57. A Fisher test was applied, and the difference proved to be non-significant over the four years of integration. The main objective here was not to degrade the ADT fields since RODAS is a multivariate assimilation scheme, meaning that the calculation of objective analyses for one variable directly impacts the other state variables of the model. This concern has been well-discussed in studies by Oke et al. [71] and Martin et al. [77], where the authors reported degradations in variables not assimilated and at depth. The U and V velocity components were also evaluated as four-year averages across all 32 layers, showing very small variation between experiments. These variables are much more sensitive to the assimilation of altimetric data, and it is desirable for SST assimilation to have minimal impact on their variability since their corrections are more strongly influenced by geostrophic balances and atmospheric forcing.

### 3.3 Brazil current dynamics

The BC, by transporting warm tropical waters to higher latitudes along the eastern coast of South America, maintains a strong positive correlation with SST. The assimilation of temperature data directly influences the model’s representation of the BC, improving the characterization of regional circulation and its variability. Furthermore, the variability of western boundary currents is linked to large-scale climate phenomena such as ENSO (El Niño–Southern Oscillation) and the AMOC [48], as well as to the formation of extratropical storms [78], which can alter their intensity, extent, and position. When the BC intensifies, there is an increase in the advection of warm waters, affecting atmospheric processes such as convection and the formation of extratropical cyclones.

The first row of Fig 6 shows the meridional velocity component at 28°S. It is observed that the assimilation of SST resulted in an attenuation of the BC core velocities compared to the Free experiment. In Free, the BC core exhibited a larger extension with velocities often exceeding 0.4 m/s. In contrast, in the RODAS_SST experiment, both the extension of the core and the velocities were reduced. This reduction makes the results more consistent with estimates from the literature, such as the in situ observations reported by Rocha et al. [79], who found an average core velocity of 0.34 m/s at 27.9°S. The reduced BC core velocities in the assimilated experiment reinforce the importance of assimilation in studies of western boundary currents.

**Fig 6:**
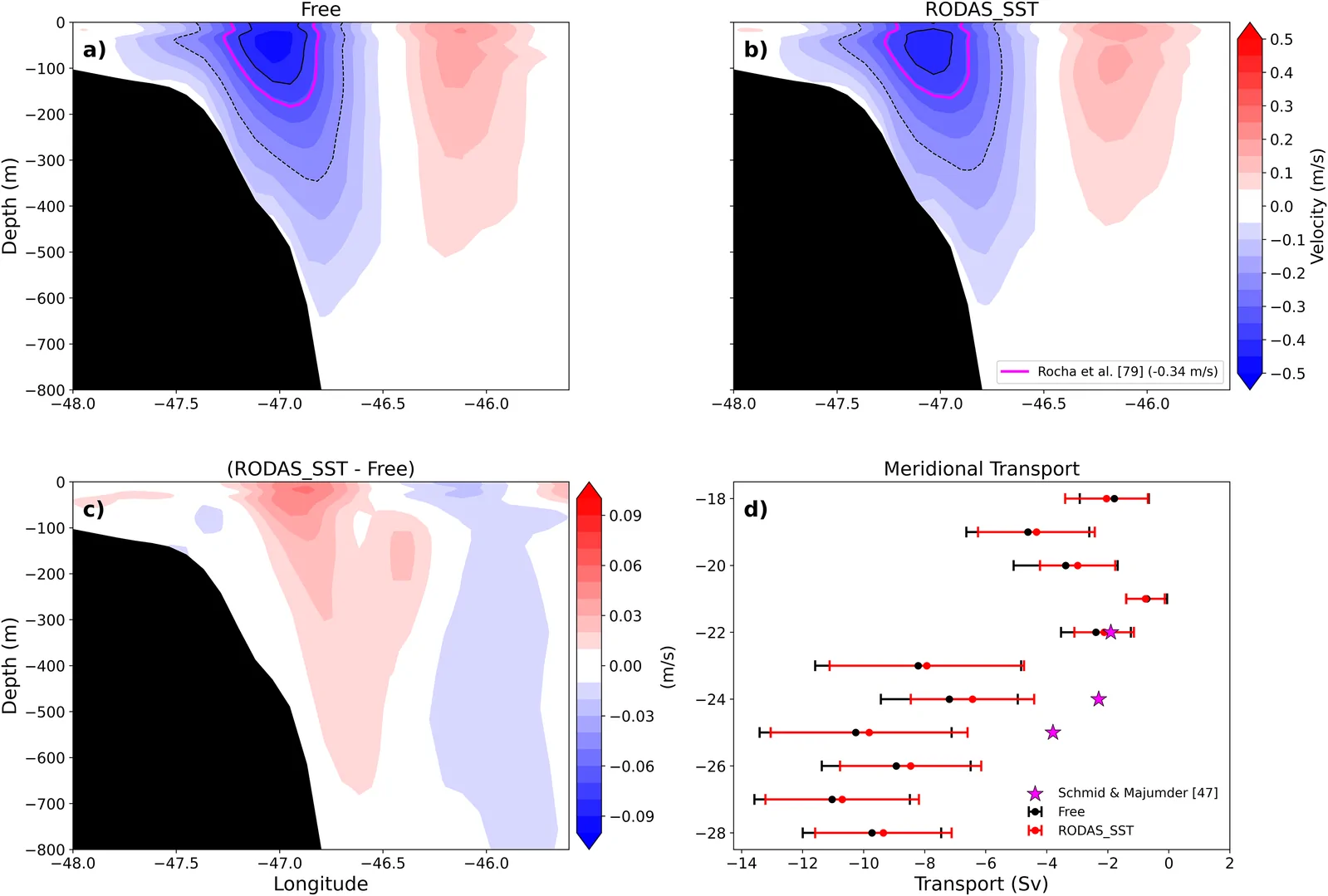
Meridional velocity component (m/s) at 28°S for the Free run (a) and the RODAS_SST experiment (b). Black dashed and solid lines indicate velocity values equal to −0.2 m/s and −0.4 m/s, respectively. The solid magenta contour highlights the −0.34 m/s velocity, corresponding to the average core velocity observed by Rocha et al. [79]. (c) Difference in meridional velocity between the assimilation experiment and the free run (RODAS_SST minus Free). (d) Latitudinal variation of the mean meridional transport (Sv) of the Brazil Current. Horizontal bars denote the standard deviation. Magenta stars represent observational transport estimates from Schmid and Majumder [47].

Schmid & Majumder [47] estimated the BC transport between 1993 and 2015 using Argo data combined with SSH from AVISO (Argo & SSH) and HYCOM+NCODA (HYCOMN) outputs. The transports calculated for Free and RODAS_SST largely remained within the deviation ranges of the HYCOMN estimates, and the intensification of the BC occurred earlier compared to the transports derived from Argo & SSH (Fig 6D). In the range corresponding to our study region, between 20°S and 27°S, the minimum and maximum transport values estimated by Schmid & Majumder [47] from Argo & SSH were 1.3 Sv and 5.6 Sv, respectively, while HYCOM+NCODA showed a variation between 2.5 Sv and 10.5 Sv. The mean values considered for each method in this range were 2.7 ± 1.4 Sv for Argo & SSH and 6.0 ± 2.6 Sv for HYCOM. In our case, the Free run showed 3.4 ± 1.7 Sv at 20°S and 11.0 ± 2.5 Sv at 27°S, while RODAS_SST obtained 3.0 ± 1.2 Sv at 20°S and 10.7 ± 2.5 Sv at 27°S. For the range between 20°S and 27°S, the Free run presented an average of 6.51 ± 2.9 Sv, and RODAS_SST 6.15 ± 2.9 Sv.

The transport values from the Free run were generally stronger than those from RODAS_SST. The assimilation of SST tended to attenuate the BC transport, which can be considered positive, since the transports simulated by ROMS were slightly higher than those presented by HYCOMN over much of the domain. This attenuation of the BC transport was statistically significant (p < 0.01), bringing the regional circulation estimates closer to the observational ranges described by Schmid & Majumder [47]. Specifically, at 22°S, 24°S, and 25°S (Fig 6D), where observational estimates of the authors indicate transports of 1.9, 2.3, and 3.8 Sv respectively, the RODAS_SST experiment reduced the overestimated flow of the Free run, yielding results more consistent with these in situ measurements.

A possible explanation for this positive effect was discussed by Goes et al. [48] and lies in the reduction of vorticity in the BC region, which could be verified in the assimilation experiment (graphs not shown). The calculation of BC transport can be influenced by mesoscale activity in the region. Thus, it is possible that nonlinear interactions between eddies and meanders in the RODAS_SST experiment contributed to a reduction in BC velocity and transport values. These interactions may result from changes in the thermal structure of the water column, in the MLD, or direct adjustments in the velocity components due to analysis increments. Furthermore, the analyzed period corresponds to a seasonal transition phase, which may present reduced transport compared to annual means. The results obtained demonstrated that the new system reproduces patterns consistent with previous studies while highlighting subtle differences related to the assimilation approach and parameterizations used by different models.

### 3.4 Ocean energy comparison

SST assimilation is fundamental for oceanic energy studies, directly influencing heat content (HC) and fluxes at the ocean-atmosphere interface (Chen et al. [80], Kerry et al. [81]). In the Southwest Atlantic, previous studies have demonstrated the critical role of data assimilation in correcting the regional thermal structure and energy distribution. For instance, De Paula et al. [18] showed that data assimilation within the ROMS framework improved the representation of mesoscale eddies and thermal gradients at the São Paulo plateau, directly impacting local heat distribution. Similarly, Tanajura et al. [32] highlighted substantial improvements in the upper-ocean thermal balance and HC estimates in the South Atlantic using the RODAS system. Building upon these regional findings, our results demonstrate that even when restricted to surface observations, the cost-effective EnOI scheme is able to project surface information into the model’s subsurface layers, refining the regional HC.

Fig 7 shows the spatial distribution of the mean absolute error (MAE) of heat content (0–300 m) between the model experiments and the GLORYS reanalysis. The maps reveal coherent large-scale patterns along the southwestern Atlantic. The largest errors are mainly found in areas of strong mesoscale activity, especially along frontal regions and where the bathymetry is complex, such as near the Vitória–Trindade Ridge. For January, the MAE decreased from 0.76 GJ/m² in the Free run to 0.65 GJ/m² in the assimilated run, representing an improvement of approximately 15%. In July, the reduction was even stronger, from 0.54 GJ/m² to 0.40 GJ/m², corresponding to a 25% decrease. The spatial error reduction achieved by the RODAS_SST experiment was found to be highly statistically significant across the domain for both seasons (p < 0.001), demonstrating that the surface assimilation dynamically constrained the integrated thermal structure of the upper ocean.

**Fig 7:**
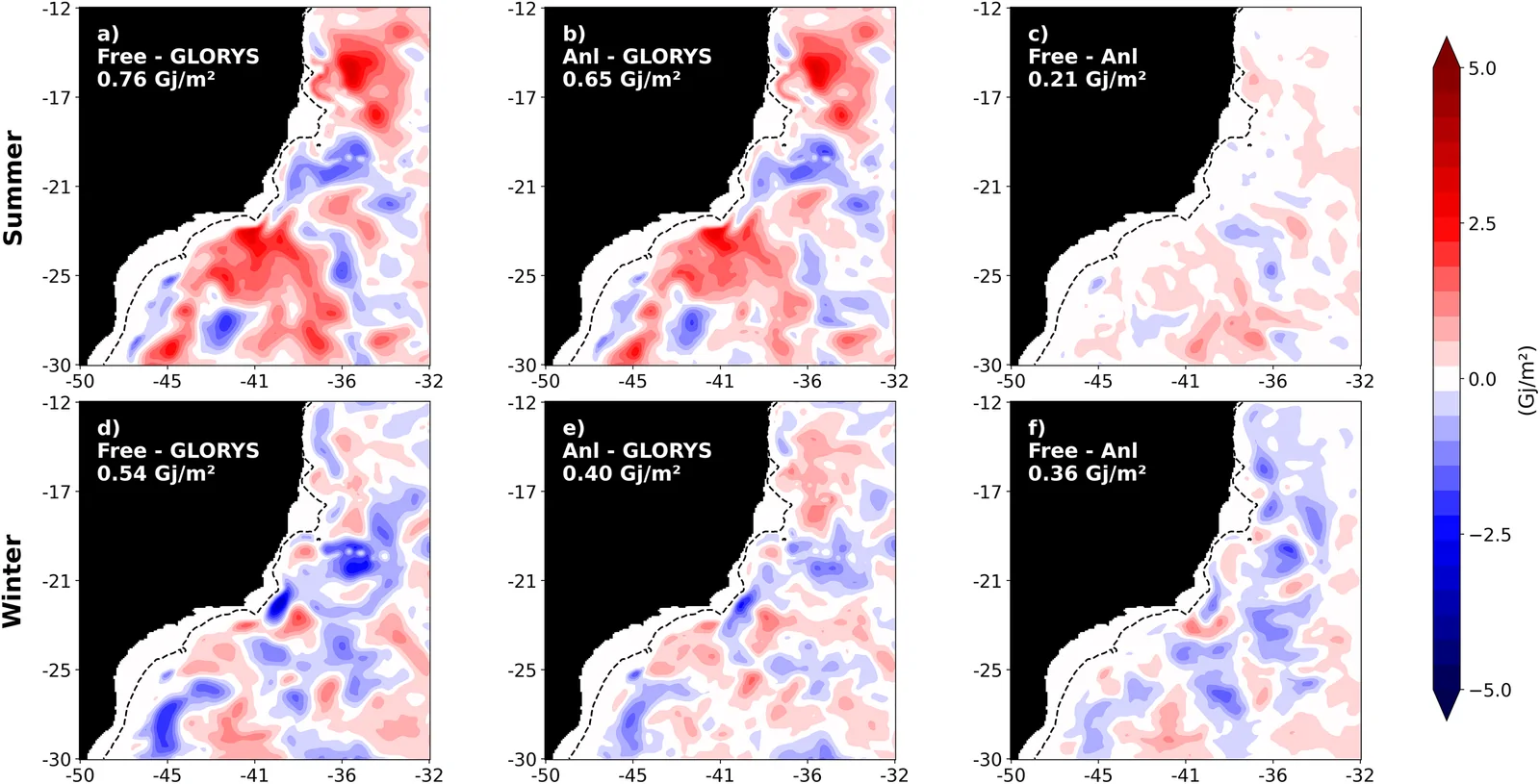
Spatial distribution of heat content differences (GJ/m²) integrated from 0 to 300 m depth for Summer/January (top row: a, b, c) and Winter/July (bottom row: d, e, f). The columns correspond to the differences between the unassimilated run and reanalysis (Free – GLORYS; a, d), the assimilated run and reanalysis (Anl – GLORYS; b, e), and the difference due to spatial impact of the assimilation (Free – Anl; c, f). The spatially averaged Mean Absolute Error (MAE) is explicitly displayed over the continent in each panel. The black dashed line indicates the 300 m isobath.

Taking into account that we only assimilated surface observations, these findings are consistent with Kerry et al. [81]‘s. The largest improvements occurred near the BC system, where strong horizontal temperature gradients prevail, suggesting once more that SST assimilation can correct the position and intensity of mesoscale meanders and eddies. Overall, the assimilation of SST within the RODAS_SST system led to substantial improvements in the representation of the upper-ocean HC, confirming the scheme was well adapted and the effectiveness of surface temperature assimilation in enhancing the vertical thermal structure.

Negative heat fluxes indicate the export of heat to the atmosphere, leading to ocean cooling and subsequent energy release at higher altitudes. Figs 8A and 8B demonstrate that both LH and SH were consistently negative throughout the integration period. However, the analysis exhibited higher absolute values compared to the Free run, suggesting a greater intensity of the predicted evaporation rate. Fig 8C and 8D reveal that the most significant differences between the experiments occurred during the winter months, affecting the entire oceanic domain. The temporal alterations induced by the RODAS_SST analysis relative to the Free run were statistically significant for both the latent heat flux (p < 0.001) and the sensible heat flux (p < 0.001).

**Fig 8:**
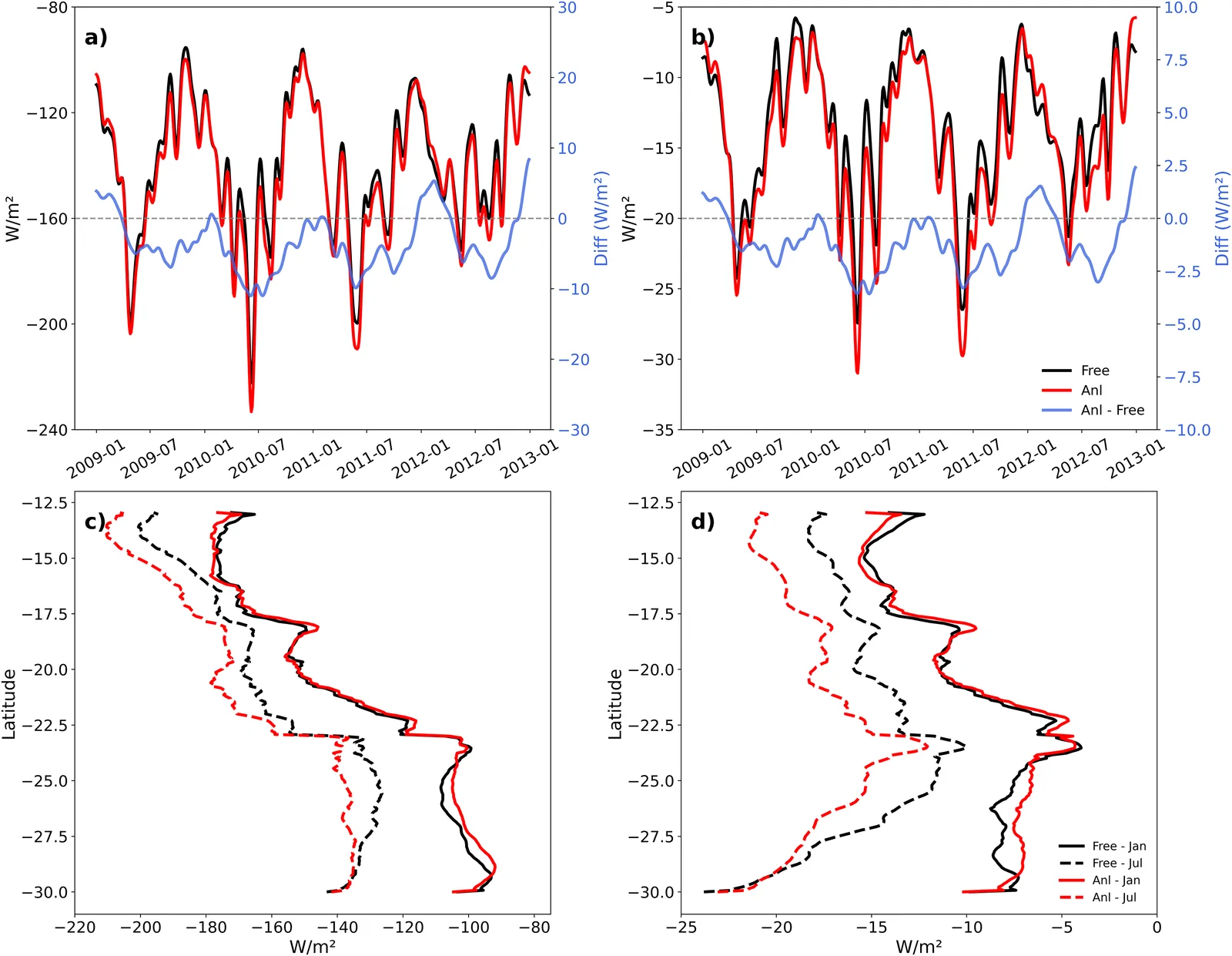
Time series of the spatially averaged latent (a) and sensible (b) heat fluxes (W/m²) for the Free run (black lines) and the assimilation experiment (Anl, red lines). The blue lines, plotted on the secondary y-axis, represent the difference between the assimilation and the free run (Anl minus Free), with a horizontal dashed gray line indicating the zero reference. Latitudinal profiles of the zonally averaged latent (c) and sensible (d) heat fluxes comparing the Free run (black) and the assimilation experiment (red) during austral summer (January, solid lines) and austral winter (July, dashed lines).

The discrepancies are closely related to the correction of the negative temperature bias observed in the Free run (as shown in Fig 1F), which was achieved through the assimilation of OSTIA data, given that atmospheric forcings remained identical in both experiments. Therefore, it can be suggested that coupled systems using ocean models without data assimilation, even if well-configured, may occasionally provide unrealistic boundary conditions for climate forecasting and studies of extreme events such as tropical cyclones and storm surges. For instance, indices such as the Genesis Potential Index [82,83] rely on the temperature contrast between the sea surface and the overlying atmosphere to estimate cyclogenesis potential, thus, biases in SST can directly degrade hurricanes forecast skills. In this sense, the improvements in upper-ocean thermal structure obtained from SST assimilation [80,81] reinforce the importance of accurate surface and subsurface temperature fields for reliable estimates of oceanic heat content. Consequently, assimilation systems such as RODAS_SST not only enhance the realism of the modeled HC but also contribute to better coupling with atmospheric models, supporting more skillful forecasts of high-impact events while maintaining low computational costs.

Fig 9 shows the spatial distribution of the monthly mean LH and SH heat fluxes for January and July, as well as their differences between the Free and assimilated experiment. The maps illustrate the typical seasonal pattern of ocean–atmosphere heat exchange along the southwestern Atlantic. Negative values correspond to heat release from the ocean, whereas positive values indicate heat gain by the ocean. The LH flux panels (Fig 9A, 9B, 9G and 9H) are predominantly negative for both experiments, except in shallow shelf areas such as the Abrolhos region (18°S) and the Cabo Frio upwelling region (~22°S). A strong agreement is observed between the RODAS_SST analysis results and those from independent data such as OAFLUX, presented by Yu et al. [55]. SST assimilation helped to refine the LH flux pattern, with the −100 W/m² isoline (Fig 9B) aligning more closely with the position shown in the OAFLUX product and the −180 W/m² isoline in Fig 9H aligning better with the authors’ winter panel values. For SH maps (Fig 9D, 9E, 9J and 9K), the January distribution pattern in the analysis (Fig 9E) closely resembles that presented by Yu et al. [55]. In July, the analysis pattern (Fig 9K) is similar to that described by Pezzi et al. [62] during SACZ events, which predominate in the summer months. Investigating the causes of this similarity may represent a promising area for future studies. The spatial error reductions achieved by the RODAS_SST analysis relative to the Free run were statistically significant across the domain for both evaluated seasons (p < 0.001). Correspondingly, the temporal alterations in the domain-averaged fluxes were also highly significant (p < 0.001). The statistical results from Figs 6–7 and 9 confirm that the surface thermal corrections propagated downwards, modifying the turbulent heat exchange parameterizations throughout the four-year integration period.

**Fig 9:**
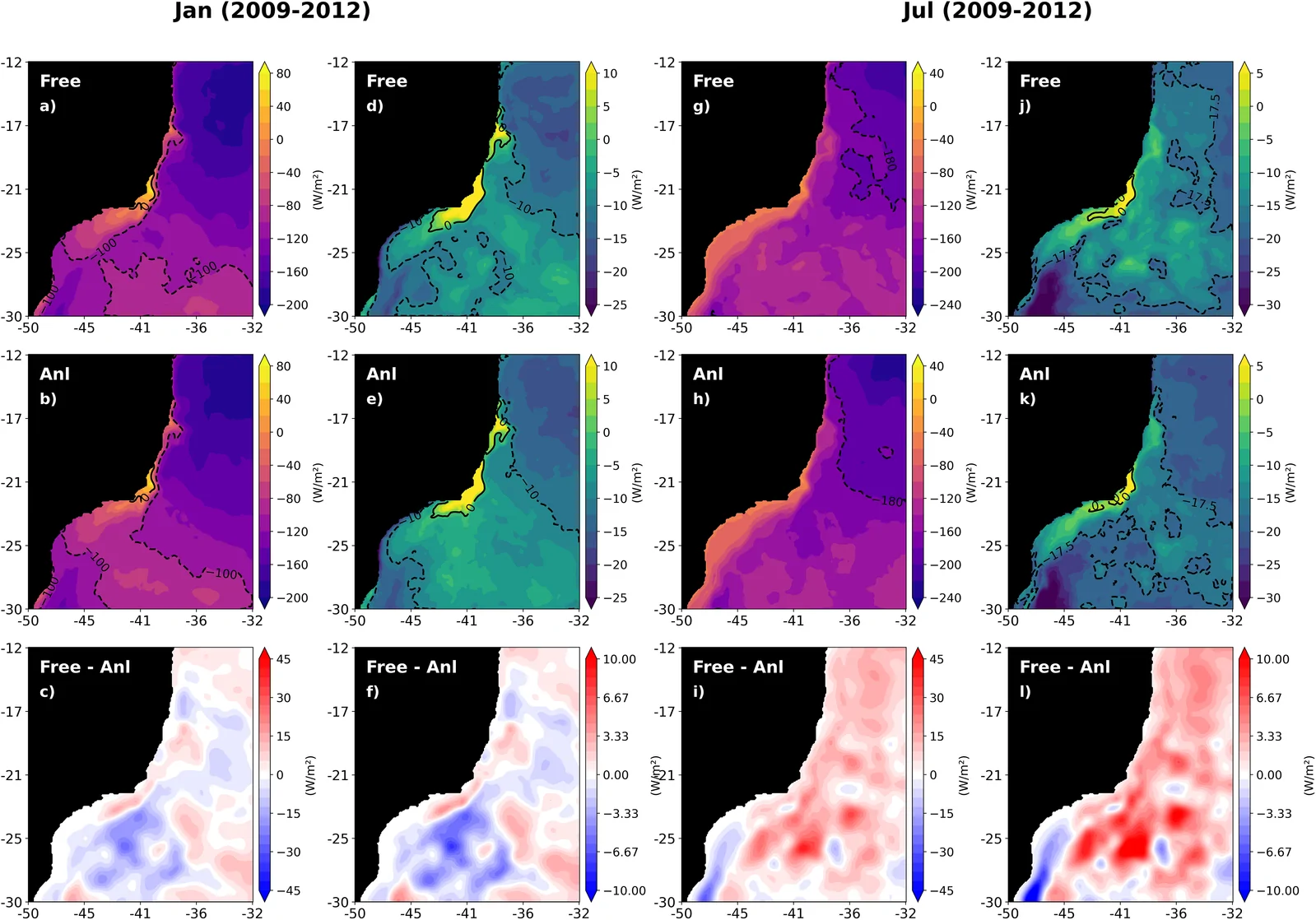
Monthly means of latent heat (LH) flux (a, b, g, h), sensible heat (SH) flux (d, e, j, k) and the difference between the two heat fluxes (Free – Anl)(c, f, i, l) for January and July. These monthly mean fields are constructed over the 4-year (2009-2012) analysis period. Negative fluxes indicate heat release from the ocean while positive fluxes indicate heat gain by the ocean. All plots are in W/m².

Seasonal differences between the experiments are notable. In January, there was a balance between positive and negative fluxes, with the Free experiment indicating greater heat export to the atmosphere (Fig 9C), especially between 45°W and 37°W and 24°S to 30°S, with differences ranging from 20 to 30 W/m² for LH and around 5–8 W/m² for SH. Clayson and Bogdanoff [84] examined the effects of diurnal SST variation and reported that differences of around 10 W/m² for SH and 60 W/m² for LH can result from 1°C variations in the SST field. The results presented here are, therefore, consistent with those described by the authors. In July, the analysis showed higher magnitudes than the Free run, suggesting a significant heat export to the atmosphere not accounted for in the Free experiment, except for the southwestern zone of the domain (Fig 9I and 9L). This likely indicates a signal of the penetration of the Malvinas Current into the study region. Differences exceeded 30 W/m² for LH and frequently surpassed 10 W/m² for SH, again, raising concerns about the use of free runs – even with relaxation, for long climate studies or forecasting extreme events such as the recent Hurricane Milton in Florida.

## 4. Final remarks and conclusion

The primary goal of this study was to initiate the development of a cost-effective data assimilation system for the ROMS model. As a first step, we implemented SST assimilation from OSTIA data. The results demonstrated that the assimilated experiment significantly reduced biases and errors relative to observations compared to the Free run. In some metrics, the ROMS+RODAS configuration outperformed the GLORYS reanalysis, highlighting the potential of this lightweight system for regional modeling and forecasting.

While SST assimilation with RODAS yielded beneficial corrections, it also introduced degradations beneath the MLD due to allowing surface-derived increments to propagate almost freely to deeper layers. Our findings emphasize the critical need for carefully designed vertical localization in regional domains. Consequently, the next iteration of the RODAS framework will confine SST increments strictly to the MLD and eliminate temperature-salinity cross-covariances to address these observed subsurface degradations.

It also is important to emphasize that this study represents a foundational assessment of the RODAS integration into ROMS. At this stage, the indirect impacts of assimilation on subsurface structures and regional dynamics, such as the Brazil Current variability, are primarily driven by the static background error covariances of the EnOI scheme. Therefore, a more thorough dynamical interpretation of these processes is reserved for future studies, when subsurface observations — such as Argo profiles — will be actively assimilated to better constrain the three-dimensional mass and energy fields.

Regarding computational efficiency, the use of the EnOI scheme in RODAS proved crucial for the feasibility of this study. As discussed previously, our quantitative estimates demonstrated that while the 4-year integration required only a few days of continuous computation for the assimilation steps using EnOI, equivalent experiments using advanced methods like 4DVar or a 90-member EnKF would have taken several months to years under the same hardware constraints. Therefore, the ROMS+RODAS coupled system provides a highly cost-effective and operationally feasible alternative for regional ocean forecasting, particularly for research groups with constrained computational resources.

To overcome the challenges of limited observation data, future implementations will expand to include the assimilation of sea surface salinity, altimetric and SWOT data, and in situ Argo profiles to further optimize performance. Once refined, our ultimate goal is to make the ROMS+RODAS system freely available with a simple and intuitive interface, aiming to democratize access to advanced, low-cost ocean modeling technologies and improve short-term predictability for extreme events such as marine heatwaves and tropical cyclones [85].
